# Contributions of the Simplified Competency Management Model to a Municipal Health Secretariat*

**DOI:** 10.1590/1518-8345.3385.3429

**Published:** 2021-07-02

**Authors:** Alessandro Albini, Aida Maris Peres, Maria de Lourdes de Almeida

**Affiliations:** 1 Prefeitura Municipal de São José dos Pinhais, Secretaria Municipal de Saúde, São José dos Pinhais, PR, Brazil.; 2 Universidade Federal do Paraná, Curitiba, PR, Brazil.; 3 Universidade Estadual do Oeste do Paraná, Foz do Iguaçu, PR, Brazil.

**Keywords:** Health Management, Public Health, Public Health Nursing, Professional Competence, Employee Performance Appraisal, Continuing Education, Gestão em Saúde, Saúde Pública, Enfermagem em Saúde Pública, Competência Profissional, Avaliação de Desempenho Profissional, Educação Continuada, Gestión en Salud, Salud Pública, Enfermeria em Salud Pública, Competencia Profesional, Evaluación del Rendimiento de Empleados, Educación Continua

## Abstract

**Objective:**

to present the contributions of the Simplified Competency Management Model in a municipal health secretariat.

**Method:**

research of integrated mixed methods of exploratory-descriptive type. The model was applied in a southern Brazilian city, in the following stages: documentary, questionnaire, mapping of gaps and educational proposal.

**Results:**

in the first stage, after documentary research, a total of 14 general core competences were described and a questionnaire with specific core competences was chosen, with confirmation of correlation among them; in the second stage, the importance and expression competence at work degrees were obtained, after the questionnaires were filled out by 74 municipal public health managers; in the third one, a formula was adopted for the training priority degree and its classification; the fourth stage presented an educational proposal for the development of one of the competences with the highest priority degree.

**Conclusion:**

the model brings contributions by describing general core competences, after documentary research; carrying out the correlation between a questionnaire, containing specific core competences with the general ones; by mapping gaps; and by the proposal of learning trails for the development of competences.

## Abstract (pt)

**Objetivo:**

apresentar as contribuições do Modelo Simplificado de Gestão por Competências em uma secretaria municipal de saúde.

**Método:**

pesquisa de métodos mistos integrados do tipo exploratório-descritivo. Aplicou-se o modelo em município sul-brasileiro, nas etapas documental, questionário, mapeamento de lacunas e proposta educacional.

**Resultados:**

na primeira etapa, após levantamento documental, foram descritas 14 competências essenciais gerais e escolhido um questionário com competências essenciais específicas, sendo realizada confirmação de correlação entre elas; na segunda, obteve-se o grau de importância das competências e o grau de expressão da competência no trabalho, após preenchimento dos questionários por 74 gestores da saúde pública municipal; na terceira, adotou-se fórmula para o grau de prioridade de capacitação e sua classificação; a quarta etapa apresentou proposta educacional para o desenvolvimento de uma das competências com maior grau de prioridade.

**Conclusão:**

o modelo traz contribuições pela descrição de competências essenciais gerais, após levantamento documental, pela realização da correlação entre um questionário contendo competências essenciais específicas com as competências essenciais gerais, pelo mapeamento de lacunas e pela proposta de trilhas de aprendizagem para o desenvolvimento de competências.

## Abstract (es)

**Objetivo:**

presentar los aportes del Modelo Simplificado de Gestión por Competencias en una secretaría municipal de salud.

**Método:**

investigación de métodos mixtos integrados de tipo exploratorio-descriptivo. El modelo se aplicó en un municipio de la región Sur de Brasil, en las etapas: documental, cuestionario, mapeo de brechas y propuesta educativa.

**Resultados:**

en la primera etapa, después de un examen documental, se describieron 14 competencias esenciales generales y se eligió un cuestionario con las competencias esenciales específicas, con la confirmación de la correlación entre ellas; en la segunda, se obtuvo el grado de importancia de las competencias y el grado de expresión de la competencia en el trabajo, después de que los cuestionarios fueron rellenados por 74 gerentes municipales de salud pública; en la tercera, se adoptó una fórmula para el grado de prioridad de capacitación y su clasificación; la cuarta etapa presentó una propuesta educativa para el desarrollo de una de las competencias con mayor grado de prioridad.

**Conclusión:**

el modelo trae aportes por la descripción de las competencias esenciales generales, después del examen documental, por la correlación entre un cuestionario que contiene las competencias esenciales específicas con las competencias esenciales generales, por el mapeo de brechas y por la propuesta de itinerarios de aprendizaje para el desarrollo de competencias.

## Introduction

Competency management is relatively new in Brazil, but its importance has been increased, being considered synonymous with good management practice^(^ 1 ^)^. Since the promulgation of Decree nº 5707/2006, which consolidated management by competences as one of the models to be followed by the federal administration, there has been a search and application of new models in the perspective of management by competencies for the public sector^(^ 2 ^)^. 

Competency management models are present in the public and private sectors of many countries, but several of them are complicated and conceptually focus on the needs of the past and present. The counterpoint would be dynamic Simplified Competency Management Model focused on knowledge, competences and behaviors that will be essential for managers in the future^(^ 3 ^)^. Competency management is also applied in countries that have national health systems, such as Canada, which has been working with core public health competences since 2008^(^ 4 ^)^.

The Association of Schools of Public Health in the Europe Region published its first edition of the European List of Core Competences for the Public Health Professional in 2006^(^ 5 ^)^. The Association is composed by health schools from countries such as Denmark, Belgium, Switzerland, France, United Kingdom, Netherlands, Poland, Serbia, Hungary and others, presenting the fifth edition of the European List of Core Competences in 2018^(^ 6 ^)^.

Generally, core competencies provide parameters for the execution of public health services, as they deal with access to health services, surveillance, disease and damage prevention, health promotion and protection^(^ 4 ^)^. Specifically in the United Kingdom, the document Public Health Skills and Knowledge Framework 2016 addresses the need for a competence structure to address issues such as: result, description of activities and functions of professionals, professional development, educational curriculum and description of functions in public health^(^ 7 ^)^.

In 2013, in Latin America, the Pan American Health Organization (PAHO) issued a document named *Competencias Esenciales en Salud Pública: Un Marco Regional para Las Américas* (MRCESP)^(^ 8 ^)^, an instrument that defines essential knowledge, skills and attitudes for the public health workforce. It also recognizes that the guarantee of compliance with public health obligations depends on competent managers, even with insufficient training offers^(^ 8 ^)^.

In Brazil, the Unified Health System - *Sistema Único de Saúde* (SUS) demands from its managers local and regional commitments, participation in various councils, creation and compliance with management instruments, in addition to the constant evaluation of inspection and social control bodies. However, some managers are not prepared to assume the management function, even though they have excellent academic credentials^(^ 9 ^)^.

Core competencies improve public health by contributing to the development of teamwork, capacity for situational analysis, planning and improvement of health services based on evidence and focused on the population in an equitable and ethical way^(^ 4 ^)^. In the development of competences, it is assumed the balance between the interests and needs of the organization and the individual with their knowledge, skills and attitudes. Thus, the consequence of investment in knowledge adds value, not only in the excellence and sustainability of the organization, but also in the social value of the individual^(^ 10 ^)^. 

Due to the complexity of this theme, it is considered that the presentation of models that correlate specific and general competencies through a mapping capable of pointing out their priority degree and educational strategies for the development of public health manager competences, is still a gap of knowledge to be filled. Thus, it is assumed that the health system can also not only benefit from the description of core competences for public health, but also develop new technologies and tools^(^ 11 ^)^ that present how competency management can be implemented.

Therefore, this study aimed to present the contributions of a Simplified Competency Management Model (SCMM)^(^ 1 ^)^ to the managers of own public health services of a Municipal Health Secretariat (MHS).

## Method

Mixed methods research, exploratory descriptive type, which combines qualitative and quantitative approaches as a classification criterion, based on its objectives^(^ 12 ^)^. 

In the presentation of the SCMM contributions, a qualitative approach was used during the documentary stage of the SCMM, in which the meaning of words and phrases were sought in the study of the official documents found, indicating the vision of the future, mission, strategic objectives, management reports, status of municipal civil servants and performance indicators^(^ 1 ^)^.

The quantitative approach highlighted the contributions of the SCMM with the mapping of gaps in its second stage, performed with multivariate analysis of the data through statistical techniques, such as the analysis of main components and internal consistency^(^ 1 ^)^. In the third stage, for the internal consistency tests of data collection, we used the Cronbach' alpha coefficient per Domain after Main Component Analysis (MCA), using the statistical software Predictive Analytics Software (PASW Statistics) 18.0.0, formerly named Statistical Package for the Social Sciences (SPSS^®^).

This study was carried out between October 2017 and June 2018 at the MHS of São Jose dos Pinhais, located in the state of Paraná, southern Brazil, with approximately a total of 320 thousand inhabitants and its own municipal public health network, comprising 46 services (Hospital and Municipal Maternity, Psychosocial Care Centers, Emergency Care Unit, Specialty Center, Mobile Emergency Care Service, Municipal Laboratory, Zoonosis Surveillance Center, Primary Care Pharmacies, Special Pharmacy and Primary Health Centers), with approximately 2 300 civil servants, of which 91 are managers.

Managers are called directors, heads or coordinators by means of a paid function, in the case of statutory employees, or by commissioned position, indicated by technical or political party criteria by the Municipal Health Secretary and endorsed by the Executive Chief. The inclusion criteria were based on the typicality of the position that public health managers of MHS own services exercise (Direction, Head or Coordination). We included individuals with responsibility for directing, heading or coordinating a department, division or public health service of MHS, who exercised decision-making, mediation, planning, control and evaluation activities, regardless of gender, age, education, professional training, if effective or commissioned server, time in office or time of management experience.

The definition of competence used in this study was based on an author^(^ 13 ^)^ who proposes the following: "competence is knowing how to act responsibly and recognized, which implies mobilizing, integrating, transferring knowledge, resources and skills, which add economic value to the organization and social value to the individual". Thus, organizational competence was disseminated internationally with the term core competency^(^ 13 ^)^, which encompasses a set of skills or aptitudes that unites individual efforts (merging expertise with the new) through harmony, communication, commitment and work organization in its various functions, which finally have positive results. 

**Figure 1:**
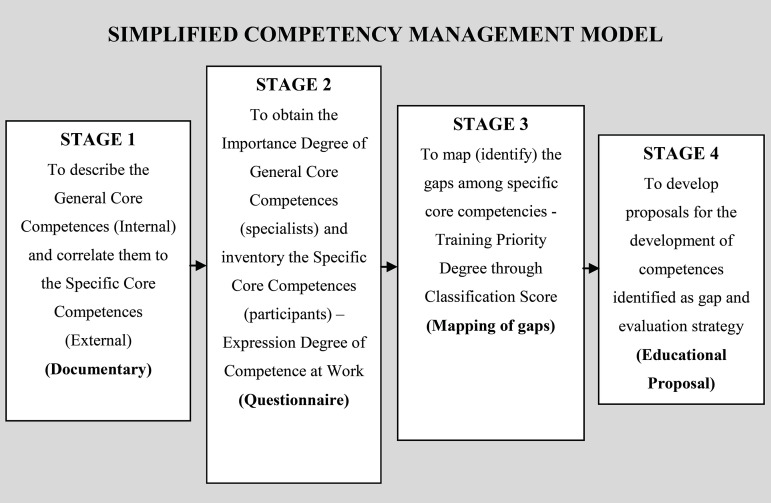
Simplified Competency Management Model

So, it was proposed to apply a SCMM with four stages: documentary, questionnaire, mapping of gaps and educational proposal (Figure 1).

In the first stage of the SCMM, documentary data collection took place in October and November 2017. The sources were the official documents of the Municipal Government and MHS, which addressed attributions or job descriptions related to the institution management and organizational strategy. These documents (regulations, rules, statutes, ordinances, action plans and management reports), with potential for interpretation and description of content, were related to the competencies of managers^(^ 1 ^)^.

After studying the official documents, the general core competences of MHS were described, indicating the composition of an expected performance or behavior of action by means of a verb or an action object accompanied by a condition and a criterion (quality standard)^(^ 14 ^)^.

Then, there was a correlation between the general core competences described for MHS and the specific core or external competences, understood as valid for the local need, choosing the document of the PAHO MRCESP^(^ 8 ^)^.

The second stage of the SCMM was applied for data collection through a semi-structured self-assessment questionnaire, translated and adapted to the Brazilian context^(^ 15 ^)^, based on the MRCESP^(^ 8 ^)^.

The questionnaire consists of response levels of five predefined, divalent, asymmetric interval points - Likert scale of five points - presented in a total of 56 questions or core competences divided into six Domains: Domain 1 - Health Situation Analysis; Domain 2 - Risk and Damage Surveillance and Control; Domain 3 - Health Promotion and Participation; Domain 4 - Policies, Planning, Regulation and Control; Domain 5 - Equity in Access and Quality in Individual and Collective Services; and Domain 6 - International and Global Health^(^ 8 ^)^.

The results of the structured questions (specific core competences) are presented by means of a central tendency measure (arithmetic mean) and a dispersion measure (standard deviation). The averages can vary between 1 and 5, with 1 being the lowest value regarding the lack of knowledge of the specific core competence and 5 representing that the competence is put into practice, with the possibility of teaching the competence to others.

In the third stage of the SCMM, the mapping of gaps was performed, resulting from the formula PD = CID (5 - CED), adapted from the one presented by Brandão(1), with the Competence Importance Degree (CID) being the result pointed out by specialist managers(15), according to the different importance understood for management^(^ 16 ^)^. The number five refers to the number of points on the Likert scale.

Thus, the Competence Expression Degree at Work (CED) was developed through the response of the managers participating in the study. So, when applying the formula, using the Microsoft Excel 2007^®^ electronic spreadsheet, it was possible to generate the numerical representation of the gap between the specific core competencies by classifying the Training Priority Degree (PD).

In the fourth stage of the SCMM, in March 2018, the educational proposal was discussed during a meeting with four representatives of the Municipal Public Health School (PHS), responsible for carrying out the educational actions, where the possibilities for developing competences were presented as gaps through learning trails^(^ 1 ^)^.

The study was submitted to the Research Ethics Committee, Opinion No. 2068137 and Certificate of Presentation of Ethical Appreciation (CAAE) No. 67007517.5.3001.0100, and met the ethical recommendations for research with human beings contained in Resolution No. 466/2012.

## Results

As a result of the first stage, after searching the physical and digital collections of MHS, the Municipal Secretariat for Planning and Economic Development and the City Hall, the documentary consultation was carried out on eighteen official documents of the city, with possible interpretation potential and description of content related to the competences of managers, a survey carried out in November 2017.

After a thorough reading of the eighteen documents found, it was understood that only three official documents stood out for their potential for interpretation and description of content related to the concept of competence. The selected documents were: Municipal Organic Law, Civil Servant Statute and the Strategic Map of the Municipal Government. In none of them the functions, attributions, qualifications, profile or responsibilities was clearly found, for any level of management (Secretariat, Directorate, Head or Coordination). However, the three official documents selected for their content served as a basis for describing 14 general core competences for MHS, as shown in Figure 2.

**Figure 2:** Description of the General Core Competences of the Municipal Health Secretariat according to official municipal documentation. São Jose dos Pinhais, PR, Brazil, 2017-2018

VERB	ACTION OBJECT	CONDITION	CRITERIA OR RESTRICTION
- Have/has (SO*); - Create (M^†^).	- Citizens/Society (SO*).	- Healthy and Safe (SO*); - Opportunities (FV^‡^); - Quality of life (FV^‡^).	- Integral and sustainable development (M^†^); - Respect (VA^§^).
GENERAL CORE COMPETENCE I: Provide citizens with integral and sustainable development to improve the quality of life with health and safety, respecting local culture and values.			
- Encourage (SO*).	- Healthy lifestyle habits (SO*).	- Sustainable (M^†^); - Quality of Life (FV^‡^).	- Commitment (VA^§^).
GENERAL CORE COMPETENCE II: Providing moments of health education with a commitment to promoting healthy habits, sustainability and quality of life to transform the social, environmental and economic conditions that impact health.			
- Encourage (SO*).	- Development of regions (SO*).	- Quality of Life (FV^‡^).	- Prioritizing the most vulnerable (SO*); - Justice (VA^§^).
GENERAL CORE COMPETENCE III: Formulate regional strategies for the development and improvement of the quality of life, prioritizing the most vulnerable regions, according to the assessment of the health situation of the local population and its trends.			
- Promote (SO*); - Maintain (M^†^).	-- Decentralization of public services (SO*).	- Integral (M^†^).	- Commitment (VA^§^); - Respect (VA^§^); - Justice (VA^§^).
GENERAL CORE COMPETENCE IV: Identify care gaps and promote the decentralization of public health services to guarantee the universality of access and comprehensive health care.			
- Improve (SO*).	- Physical structures in the critical areas of the City Hall (SO*).	- Integral (M^†^); - Prompt service (S^||^).	- Commitment (VA^§^); - Respect (VA^§^); - Justice (VA^§^).
GENERAL CORE COMPETENCE V: Coordinate the maintenance and/or expansion of the existing physical structures for the provision of health services to ensure universal access and comprehensive health care.			
- Implant (SO*); - Create (M^†^).	- Shared management of municipal public services (SO*).	- Integral (M^†^); - Prompt service (S^||^); - Improving their duties (S^||^); - Update and expand their professional knowledge (S^||^).	- Commitment (VA^§^); - Respect (VA^§^).
GENERAL CORE COMPETENCE VI: Implement the shared management of municipal public services and promote integration, commitment and respect between them and the improvement of their duties towards society.			
- Become (SO*), - Compliance (L^¶^).	- Analysis processes (SO*); - Inspections (SO*); - Acts and regulations (L^¶^); - Laws, decrees and regulations (L^¶^).	- More agile and effective (SO*); - Modern City (FV^‡^); - Good execution (L^¶^); - Prompt service (S^||^); - Improving their duties (S^||^).	- Justice (VA^§^); - Transparency (VA^§^); - Respect (VA^§^); - Innovation (VA^§^).
GENERAL CORE COMPETENCE VII: To provide health surveillance in the fulfillment of analysis and inspection processes, with justice and transparency, offering more agile, modern, innovative and effective services to promote the respect and protection of society.			
- Mejorar (OE*).	- Comunicación (OE*); - Gestión de la Información (OE*).	- Oportunidades (VF^‡^); - Efectividad en el atendimiento (E^||^).	- Transparencia (VA^§^); - Innovación (VA^§^).
GENERAL CORE COMPETENCE VIII: Communicating with citizens, offering them relevant information in clear, objective, accessible and innovative language.			
- Optimizar (OE*); - Cumplimiento (L^¶^).	gubernamental (OE*); - Actos y reglamentos (L^¶^); - Leyes, decretos y reglamentos (L^¶^).	- Calidad (VF^‡^); - Buena ejecución (L^¶^); - Cuidado y Dedicación (E^||^); - Moralidad administrativa (E^||^).	- Compromiso (VA^§^); - Ética (VA^§^); - Respeto (VA^§^); - Transparencia (VA^§^).
GENERAL CORE COMPETENCE IX: Manage the efficiency of government spending and the quality of municipal public services with zeal, legality, commitment, ethics, respect, transparency and administrative morality.			
- Aumentar (OE*).	- Participación de recursos externos en las inversiones municipales (OE*).	- Oportunidades (VF^‡^).	- Compromiso (VA^§^).
GENERAL CORE COMPETENCE X: Formulate strategies for establishing partnerships, technical cooperation and attracting external resources for investments in municipal public services.			
- Develop (SO*).	- Leadership (SO*).	- Opportunity (VF^‡^); - Update and expand their professional knowledge (S^||^).	- Commitment (VA^§^).
GENERAL CORE COMPETENCE XI: Formulate strategies for the development and prominence of leaders among municipal employees, updating and expanding their professional knowledge.			
- Encourage (SO*).	- Good organizational environment (SO*).	- Motivation (SO*); - Spirit of cooperation and solidarity (S^||^).	- Ethic (VA^§^); - Respect (VA^§^); - Justice (VA^§^); - Transparency (VA^§^).
GENERAL CORE COMPETENCE XII: Develop motivational and conflict mediation processes to encourage cohesion, harmony and a good organizational environment at work, with ethics, respect, justice and transparency.			
- Line (SO*), - Fulfillment (L^¶^).	- Competences (SO*); - Acts and regulations (L^¶^); - Laws, decrees and regulations (L^¶^).	- Needs pointed out by the administration (SO*); - Good execution (L^¶^); - Improving their duties (S^||^).	- Commitment (VA^§^).
GENERAL CORE COMPETENCE XIII: Line the managerial competences of municipal managers to the commitment to the needs pointed out by the administration in compliance with laws, decrees and regulations.			
- Establish (SO*).	- Policies (SO*).	- Valuing people (SO*); - Know the specific legislation related to their attributions and functional life (S^||^).	- Meritocracy (SO*); - Opportunity (VA^§^); - Justice (VA^§^); - Transparency (VA^§^).
GENERAL CORE COMPETENCE XIV: Implement policies and processes of corporate education, performance evaluation and reward system supported by justice, transparency and meritocracy.			

* SO = Strategic objectives;

† M = Mission;

‡ FV = Future vision;

§ VA = Values;

|| S = Statute of Municipal Servants;

¶ L = Municipal Organic Law

Then, the 14 general core competences described for MHS were correlated to the six domains of specific (or external) core competencies of the questionnaire based on MRCESP, demonstrating that the selected (external) questionnaire, with specific, selected competences holds potential for the development of the general (internal) competences described.

Domain 1 (Health Situation Analysis)(8) was correlated to General Core Competence (GCC) III. Domain 2 (Surveillance and Risk and Damage Control)(8) to GCC VII. Domain 3 (Health Promotion and Participation)(8) to GCC I, II, VIII. Domain 4 (Policies, Planning, Regulation and Control)(8) to GCC IX, XI, XII, XIII and XIV. Domain 5 (Equity to Access and Quality in Individual and Collective Services)(8) to GCC IV and V; and, finally, Domain 6 (International and Global Health)(8) to GCC VI and X.

In the second stage of the SCMM, a questionnaire was applied between October 2017 and January 2018. Of the total of 91 managers approached, 85.71% (n=78) completed the questionnaire between 22 and 50 minutes. Four questionnaires, which were incomplete, were removed from the study to guarantee the quality of the collected data, with 81.39% (n=74) of the questionnaires being computed.

The result of the mapping of gaps in the third stage, as shown in Table 1, shows the gaps among the core competences for public health, through the PD result, are the core competences with the highest score, presented with Moderate Priority (Figure 3). Among the 56 core competencies, eight were identified as gaps.

**Table 1:** Gaps mapping - identification of the degree of training priority (PD). São Jose dos Pinhais, PR, Brazil, 2017-2018 (n=74)

Specific Core Competence	Competence Importance Degree (CID)	Competence Expression Degree at Work (CED^‡^)	Training Priority Degree (PD*) PD* = CID^†^ (5 - CED^‡^)
Domain 1	4.56	3.62	6.3
DO^§^1CO^||^1	4.62	3.57	7
DO^§^1CO^||^2	4.51	3.36	7
DO^§^1CO^||^3	4.52	3.72	6
DO^§^1CO^||^4	4.55	3.62	6
DO^§^1CO^||^5	4.74	3.59	7
DO^§^1CO^||^6	4.52	3.58	6
DO^§^1CO^||^7	4.39	3.93	5
DO^§^1CO^||^8	4.67	3.49	7
DO^§^1CO^||^9	4.54	3.73	6
Domain 2	4.56	3.11	8.6
DO^§^2CO^||^1	4.66	3.22	8
DO^§^2CO^||^2	4.59	2.97	9
DO^§^2CO^||^3	4.46	3.11	8
DO^§^2CO^||^4	4.53	3.41	7
DO^§^2CO^||^5	4.51	3.23	8
DO^§^2CO^||^6	4.53	3.32	8
DO^§^2CO^||^7	4.51	3.50	7
DO^§^2CO^||^8	4.55	3.20	8
DO^§^2CO^||^9	4.64	3.27	8
DO^§^2CO^||^10	4.39	2.93	9
DO^§^2CO^||^11	4.56	2.82	10
DO^§^2CO^||^12	4.66	2.80	10
DO^§^2CO^||^13	4.64	2.89	10
DO^§^2CO^||^14	4.66	2.81	10
Domain 3	4.52	3.47	6.9
DO^§^3CO^||^1	4.47	3.73	6
DO^§^3CO^||^2	4.52	3.61	6
DO^§^3CO^||^3	4.51	3.14	8
DO^§^3CO^||^4	4.47	3.77	5
DO^§^3CO^||^5	4.66	3.30	8
DO^§^3CO^||^6	4.46	3.46	7
DO^§^3CO^||^7	4.44	3.53	7
DO^§^3CO^||^8	4.42	3.41	7
DO^§^3CO^||^9	4.45	3.39	7
DO^§^3CO^||^10	4.76	3.34	8
Domain 4	4.7	3.61	6.5
DO^§^4CO^||^1	4.78	3.54	7
DO^§^4CO^||^2	4.66	3.81	6
DO^§^4CO^||^3	4.74	3.57	7
DO^§^4CO^||^4	4.78	3.76	6
DO^§^4CO^||^5	4.58	3.59	6
DO^§^4CO^||^6	4.60	3.69	6
DO^§^4CO^||^7	4.80	3.32	8
Domain 5	4.65	3.67	6.2
DO^§^5CO^||^1	4.41	3.81	5
DO^§^5CO^||^2	4.73	3.66	6
DO^§^5CO^||^3	4.74	3.72	6
DO^§^5CO^||^4	4.56	3.46	7
DO^§^5CO^||^5	4.78	3.55	7
DO^§^5CO^||^6	4.74	3.43	7
DO^§^5CO^||^7	4.49	3.91	5
DO^§^5CO^||^8	4.73	3.78	6
Domain 6	4.65	2.97	9.4
DO^§^6CO^||^1	4.62	3.05	9
DO^§^6CO^||^2	4.67	3.12	9
DO^§^6CO^||^3	4.68	2.77	10
DO^§^6CO^||^4	4.66	2.84	10
DO^§^6CO^||^5	4.73	2.91	10
DO^§^6CO^||^6	4.69	3.03	9
DO^§^6CO^||^7	4.69	3.35	8
DO^§^6CO^||^8	4.49	2.72	10

* PD = Training Priority Degree;

† CID = Competence Importance Degree;

‡ CED = Competence Expression Degree at Work;

§ DO = Domain of the Answered Questionnaire;

|| CO = Competences of the Answered Questionnaire

**Figure 3:** Classification of the Training Priority Degree

SCORE	Training Priority Degree (PD*)
0 - 5	Null or Very Low Priority
6 - 9	Low Priority
10 - 11	Moderate Priority
12 - 20	High Priority

From this mapping, the gaps in Domain 2 (Core Competences 11, 12, 13 and 14) stand out - referring to risk management, health risk reduction, immediate response and reconstruction, all referring to disasters - and in Domain 6 (Core Competencies 3, 4, 5 and 8), in which core competencies, mapped as gaps, address transnational initiatives, global and international health.

The internal consistency of the collected data demonstrates a high coefficient for Cronbach's alpha (reliability), for data collected in all domains and second order constructs (results above 0.75).

As a result, a Classification by Score indicating the PD was also adapted, as shown in Figure 3.

As a result of the fourth stage, after meeting with the PHS team, one of the specific core competences identified with the highest PD (DO2CO11) was selected to be used as an example in the definition of content and teaching theme, through Knowledge, Skills and Attitudes (KSA)^(^ 17 ^)^, according to Figure 4.

**Figure 4:** Example of Definition of Content and Teaching Theme - Knowledge, Skills and Attitudes (KSA). São Jose dos Pinhais, PR, Brazil, 2017-2018

Selected Core Competency	KNOWLEDGES (Strategic thought) KNOW WHAT / KNOW WHY	SKILLS (Process Management) KNOW HOW	ATTITUDES (Motivation) DETERMINATION
DO*2CO^†^11 Participate in disaster risk management plans in the face of natural, technological and biological threats to mitigate their health effects.	- Know the National Civil Defense and Protection Policy; - Know the classification of disasters; - Know the means of disaster prevention - Know the main disasters that affect the city; - Know the effects of disasters on municipal health services; - Make contact and approach Civil Defense; - Know the risk areas in the city; - Know about disaster risk planning and management.	- Technique: Planning; - Interpersonal: Communication with the Municipal and State Civil Defense; - Conceptual: Intersectoral work; - Decision-making: recognize problems and opportunities; - Management: Set priorities.	Initiative; Will; Team work; Disposition; Courage; Altruism; Empathy

* DO = Domain;

† CO = Specific Core Competence

As a learning proposal, options can be individualized according to the aspirations and preferences of each manager, that is, some of the options that can compose learning trails to develop the specific core competence pointed out as a gap^(^ 1 ^)^, participate in risk management plans disasters in the face of natural, technological and biological threats to mitigate their effects (DO2CO11), are:

- Distance education course (Theme: Incident command system);
- Face-to-face lecture (Theme: Technologies and innovations for disaster prevention);
- Book (Reducing vulnerability to disasters: from knowledge to action);
- Article (The National Civil Defense and Protection Policy: disasters as a political problem);
- Videos (Vulnerability to disasters: looks at the past, present and future);
- Internet pages (National Center for Monitoring and Natural Disaster Alerts, State Coordination for Civil Protection and Defense and the Civil Defense Computerized System of the State of Paraná - Occurrence Report);
- Mentoring (Professional and pedagogical support related to disasters);
- Forum (Disaster risk prevention);
- Workshop (Comprehensive risk and disaster management);
- Specialization Course (Disaster Management in protection and Civil Defense).

Finally, as a result of the study and search for evaluation techniques to perceive the development of the specific core competence, identified as a gap after the learning path has been covered, there is a proposal for impact indicators in the categories: Level (individual or organization), Measurement Complexity (depth and or breadth), Data Collection Instrument (questionnaire or document analysis), Data Collection Source (manager or colleagues) and Observed Impacts (improving competence or advances and achievements)^(^ 18 ^)^.

Depth refers to advances in the performance of activities related to the objectives and contents taught (core competences) and impact on work. On the other hand, the amplitude refers to the positive effects in other activities not directly related to the objectives and contents taught^(^ 18 ^)^.

In the case of Level of Assessment of the impact on the individual, a questionnaire is suggested to measure the Complexity of the Measure regarding depth (first six questions) and breadth (last six questions), to be applied after the manager meets the learning path, with a total of 12 questions with answers structured on a Likert scale of agreement^(^ 18 ^)^.

In order to measure the Level of Evaluation of the impact on the organization, the Complexity of the Measure is proposed only in depth and as a Data Collection Instrument, the documentary analysis; the Data Collection Sources are the SUS Management Instruments and the Observed Impacts are the advances and achievements related to the competence developed registered in the management instruments, such as the Previous Four-monthly Detailed Report (PFDR) and the Annual Management Report (AMR).

## Discussion

Regarding the first (documentary) stage, the SCMM contribution to the finding, during the search for official documents, absence of a description of the functions, attributions, qualifications, profile or responsibilities of the managers (secretariat, director, chief or coordinator) stands out. The performance of the manager depends on clear specifications and provided by the organization, with an updated job description, covering the responsibilities and duties, by department. Thus, the job description is like a road map of where they want to be and how(19-20).

The first stage of the SCMM also provided the institution with a description of 14 unprecedented general core competencies, which enhance the implementation of organizational goals and strategies to promote motivation, monitoring, evaluation, coordination and synchrony of efforts in different services and accelerate public management decision-making processes by sharing defined objectives^(^ 21 ^)^.

Another contribution of the first stage is the correlation between the specific general core competences described for the institution (MHS) and the six Domains of MRCESP^(^ 8 ^)^, a questionnaire chosen as a data collection instrument, which contains the specific (or external) core competences. 

It is understood that to put the SCMM into practice, you can choose any questionnaire containing specific competences. However, it is recommended that a correlation be made with the general core competences so that efforts are not expended, by pointing out gaps or time in educational projects, to develop external core competences, which do not add up to what is expected by the public administration.

As a contribution of the second stage of the SCMM, there is the presentation of the evaluation of specific core competences by specialists, resulting in the CID. Thus, competencies receive individualized results, far from a unanimous ideal among all competencies for a proposition of level of acceptability, which can vary a lot or little between one competence and another, because without the evaluation, among the numerous general and specific competencies that exist, it would be difficult to discern priorities^(^ 22 ^)^.

In this same second stage, the CED is defined when the questionnaire respondent performs a self-assessment regarding the possession of the knowledge, skills and attitudes necessary to put the specific core competence into practice.

In the third stage of the SCMM, the formula adapted from the one presented by Brandão^(^ 1 ^)^ presents itself as a tool of effective contribution, because in addition to practicality and simplicity in use, it results in the PD, providing focus on specific core competencies pointed out as gaps, that is, with a higher score. Still in the third stage of the SCMM, the Classification of the PD contributes to the evaluation of the results of the applied formula, when observing that the participating managers present satisfactory results, as they presented only eight gaps (of a total of fifty-six) with the maximum score being 10 (moderate priority).

The four gaps in Domain 2 (Surveillance and Control of Risks and Damages) presented in the third stage of the SCMM address specific core competences referring to intersectoral planning for managing natural, technological, biological and post-disaster reconstruction, with the damage identification and immediate rehabilitation. The relevance for the city of the core competences, identified as gaps related to disasters, is confirmed by the report of the State Coordination of Protection and Civil Defense of the State, since in 2016 the city of this study had the highest number of occurrences in the State: 17 disasters^(^ 23 ^)^. 

Therefore, this is not a concern only in Brazil. In Africa, the World Health Organization, in partnership with public health training institutions, conducted a pilot study on the development of core competences and training curriculum on Risk and Disaster Management^(^ 24 ^)^.

The four gaps in Domain 6 (International and Global Health) deal with core competences related to transnational intersectoral initiatives to overcome inequalities, and global health, as contrasts between public health systems and their influence on health, welfare, social security and fundraising reforms, which are important to the city, which, in addition to having an international airport, is located in a vulnerable (metropolitan) region.

Regarding the statistical analysis of main components and internal consistency of the collected data, the reliability results (Cronbach's alpha coefficient) were high for all domains, even with the number of items per domain, mostly below ten questions^(^ 25 ^)^, and a 95% confidence interval level and a 5% margin of error. However, it is emphasized that the performance of statistical analysis is not mandatory for the use of the SCMM.

On the other hand, if data are collected for scientific research, it is recommended that there be at least 50 participants^(^ 26 ^)^ and that, after extracting factors with the proper rotation, sample adequacy, sphericity and factorial model tests, parallel analysis, determinant value, non-redundant residue, removal of possible outliers, and if necessary, maximum likelihood, goodness-of-fit index and adjusted goodness-of-fit index, before calculating Cronbach's alpha coefficient.

The fourth stage of the SCMM contributes by exemplifying how the specific core competence, mapped as a gap, can be decomposed into knowledge, skills and attitudes, guiding the definition of content and teaching theme for the development of competence on disasters. An educational structure is a set of competencies that takes into account performance based on educational needs, personal experience, professional role and role in planning, mitigation, response and disaster recovery^(^ 27 ^)^.

Competency-Based Education (CBE) assumes that different sets of competencies are feasible to be customized for different education needs in the health professions, favoring the flexibility of time, place or learning pace, with multiple paths^(^ 28 ^)^. The permeability of higher education, which advances to the field of professional development, has been of interest in Europe and the United States as it envisions greater potential for innovation with the activation of flexible personal learning paths^(^ 29 ^)^.

A study carried out with managers of primary health care in southern Brazil pointed out the lack of training as a reason for dissatisfaction, and permanent health education as a strategy for meaningful learning, with a methodological axis in their work processes^(^ 30 ^)^. This panorama converges to the use of the SCMM, which contributes to the systematization in sequence, from the reflection and identification of problems (identification of gaps), to the search for practical solutions after the development of specific competences.

The process of shifting the focus from the transfer of knowledge to the development of competences must result from the tuning of the theory to real work situations, seeking excellence in public health practice and reducing the distance between what is learned and what is practiced^(^ 31 ^-^ 32 ^)^, because formal education, even in public health, does not, or little, develops the core competences expected of a manager^(^ 33 ^-^ 34 ^)^.

This study proposes the development of learning paths for the development of any core competence identified as a gap. It is based on the premise that, when the expected competencies are not expressed, it is possible to manage the development of competences in facing complexities and evolving demands^(^ 35 ^)^. 

The proposal for learning paths for public management is still incipient and little known, as an alternative to training grids. As a strategy to promote the development of competences by combining the expectations of the organization with the characteristics and profile of each person, the trails can be characterized as empirical and non-systematic strategies^(^ 36 ^)^, even though they offer conveniences for organizational learning.

The formatting that can be chosen for the learning paths is vast and unlimited, relying on the resources of Information Science to organize and represent information^(^ 36 ^)^, in addition to other better known as lectures, seminars, face-to-face and distance courses, conferences, study trip, specialization course, books, manuals, articles, movies, forums, websites, videos, coaching and mentoring.

As an example of a resource, mentoring has been used for more than a decade in the framework of research in the field of health, in addition to being present in teaching situations. It is also appropriate in specific cases of work and in the development of leadership competences^(^ 37 ^)^. Mentoring, recognized and recommended as an innovative, low-cost competence development, has been implemented in health institutions, with the temporary stay of a mentor, visits by an itinerant mentor, a team of multi-professional itinerant mentors, a mentor for two health services or permanent manager-mentor^(^ 38 ^)^.

Without evaluation, the teaching-learning cycle is not closed. The contribution of the fourth stage is also related to the assessment techniques for perceiving the development of the specific core competence identified as a gap. It is considered important to evaluate the results of the impact of learning activities by the individual, in the feedback for the proper improvement of the model and for the organization, in ensuring that the efforts expended are being effective to the disposition^(^ 18 ^)^.

As limitations of this study, it is pointed out that the questionnaire used does not address financial aspects of management, and they have not been explored. Still, due to the determined validity period of the SCMM, possibly around six months^(^ 1 ^)^, there is a demand for new applications of the model for continuity of the process, since the services, contexts and their actors are dynamic.

## Conclusion

This study analyzed the contributions of a SCMM consisting of four stages (documentary, questionnaire, mapping of gaps and educational proposal) for a MHS and appears unprecedented in Brazilian public health. 

In the first stage, the SCMM made contributions by noting the lack of description of the functions, duties, qualifications, profile or responsibilities of managers (secretariat, director, chief or coordinator) in official municipal documents; exemplified how general core competencies can be described; detailed the correlation of general core competences with questionnaires containing specific (external) core competencies.

In the second stage, the SCMM made explicit contributions to the generation of the Importance and the CED through the analysis of the questionnaire results.

In the third stage, the SCMM evidenced contributions on how to generate the PD when performing the mapping of gaps through formula and Classification of the PD.

In the fourth stage, the SCMM presented contributions on how to break down a specific core competence in Knowledge, Skills and Attitudes; proposed an individualized educational intervention through Learning Trails to develop competences; proposed impact assessment techniques for the perception of competence development.

As a recommendation for future studies, it is suggested that the experts consulted to generate the Competences Importance Degree are from the service, as the importance of different competencies is based on local needs, contexts and people. Also, the performance of the gap mapping step must be separated by the different levels of managers and or by departments, which allows more specificity in the planning of the learning trails.
